# Mechanisims of asthma and allergic disease – 1091. Eosinophilia nasal impact over the lunch function tests in patients with moderate to severe persisten allergic rhinitis

**DOI:** 10.1186/1939-4551-6-S1-P87

**Published:** 2013-04-23

**Authors:** Maricruz Calva, Sandra Nora Gonzalez, Alejandra Macías, Alfredo Arias, Claudia Ivonne Gallego, Diego De Jesus García, Karla Mejía, Luis Alfredo Dominguez, Vanessa Yañez, Hilda Hernández, Lorena Rangel

**Affiliations:** 1 Centro Regional De Alergia e Inmunología Clínica, Hospital Universitario, Monterrey, Mexico; 2 Allergy and Clinical Immunology, University Hospital, Monterrey, Mexico; 3 Centro Regional De Alergía E Inmunología Clínica, Hospital Universitario "Eleuterio González", Monterrey, Mexico; 4 Regional Center of Allergy and Clinical Immunology, University Hospital, Monterrey, Mexico

## Background

To understand the impact of nasal eeosinophilia in patients with moderate to severe persistent allergic rhinitis over the lung fuction, focused on FEV1, FvC and the presence or not of reversibility Background.

## Methods

We included patients at age of 7 years or more, with diagnosis of moderateto severe persistent allergic rhinitis that were evaluated between march of 2010 and june of 2011 at our Regional Center. All patient werw submitted to an spirometry, nasal cytology and a quantitatively nasal eosinophilia measured by optical microscopy. Study design: one center, observational, descriptive and transversal**.**

## Results

90 patients were included, 73 of the patients didn´t present reversibility and 53.4% were men. The reversibility was significantly greater when associated with the presence of eosinophilia, by a quantitative analysis and by crossings analysis(P=0.004 and 0.003). The eosinophilia count by a quantitative analysis in relation to the FEV1 and the FVC did not show statistical significance (P=0.116 and P=0.49). There was no difference between the relation of the implicated aeroellergen type and the nasal eosinophilia or the reversibility. The time of evolution with the grade eosinophilia and the presence of reversibility did not showed statistical significance.

## Conclusions

The allergic rhinitis is a complex disease that involves also lower airway, finding confirmed by the presence of reversibility and a tendency to diminish the basal parameters of the spirometry. We did not found significant difference between the time of evolution neither the type of aeroallergen involved with the presence of reversibility or the amount of nasal eosinophilia.

